# A Six-Year Point Prevalence Survey of Healthcare-Associated Infections in an Italian Teaching Acute Care Hospital

**DOI:** 10.3390/ijerph17217724

**Published:** 2020-10-22

**Authors:** Andrea Gentili, Marcello Di Pumpo, Daniele Ignazio La Milia, Doriana Vallone, Gino Vangi, Maria Incoronata Corbo, Filippo Berloco, Andrea Cambieri, Gianfranco Damiani, Walter Ricciardi, Patrizia Laurenti

**Affiliations:** 1 Section of Hygiene, University Department of Life Sciences and Public Health, Università Cattolica del Sacro Cuore, 00168 Rome, Italy; andrea.gentili1989@gmail.com (A.G.); dvmedi@gmail.com (D.V.); gianfranco.damiani@unicatt.it (G.D.); walter.ricciardi@unicatt.it (W.R.); patrizia.laurenti@unicatt.it (P.L.); 2 Department of Woman and Child Health and Public Health-Public Health Area, Fondazione Policlinico Universitario A. Gemelli IRCCS, 00168 Rome, Italy; danieleignazio.lamilia@policlinicogemelli.it; 3 Medical Management, Fondazione Policlinico Universitario A. Gemelli IRCCS, 00168 Rome, Italy; gino.vangi@policlinicogemelli.it (G.V.); mariaincoronata.corbo@policlinicogemelli.it (M.I.C.); filippo.berloco@policlinicogemelli.it (F.B.); andrea.cambieri@policlinicogemelli.it (A.C.)

**Keywords:** health care associated infections, teaching acute care hospital, prevalence survey, public health, risk factors, infection control

## Abstract

Healthcare-associated infections (HAI) represent one of the most common cause of infection and an important burden of disease. The aim of this study was to analyze the results of a six-year HAI point prevalence survey carried out yearly in a teaching acute care hospital from 2013 to 2018, following the European Center for Disease Prevention and Control (ECDC) guidelines. Surgical site infections, urinary tract infections, bloodstream infections, pneumonia, meningitis, and Clostridium difficile infections were considered as risk factors. A total of 328 patients with HAI were detected during the 6-year survey, with an average point prevalence of 5.24% (95% CI: 4.70–5.83%). Respiratory tract infections were the most common, followed by surgical site infections, urinary tract infections, primary bloodstream infections, Clostridium difficile infections, and central nervous system infections. A regression model showed length of stay at the moment of HAI detection, urinary catheter, central venous catheter, and antibiotic therapy to be the most important predictors of HAI prevalence, yielding a significant adjusted coefficient of determination (adjusted R^2^) of 0.2780. This will provide future infection control programs with specific HAI to focus on in order to introduce a proper prophylaxis and to limit exposure whenever possible.

## 1. Introduction

Healthcare-associated infection (HAI) is an infection occurring in a patient during the process of care, in a hospital or other health care facilities, which was not present or incubating at the time of admission, therefore HAI can also appear after discharge [1]. HAI are among the most serious public health problems both in Europe and in Italy, representing the most frequent adverse event during care delivery and causing prolonged hospital stay, long-term disability, substantial additional costs for health systems, and unnecessary deaths [2].

Despite the existing body of evidence describing interventions that can substantially reduce the rate of HAI, a point-prevalence survey conducted by the ECDC, estimated that approximately 4 million patients acquire a HAI each year in all EU Member States and that approximately 37,000 deaths directly result from these infections, with a prevalence of patients with at least one HAI in acute care hospitals of 6.0% (country range 2.3–10.8%). The most frequently reported HAI types were respiratory tract infections (pneumonia 19.4% and lower respiratory tract 4.1%), surgical site infections (19.6%), urinary tract infections (19.0%), bloodstream infections (10.7%), and gastro-intestinal infections (7.7%), with Clostridium difficile infections accounting for 48% of the latter [3].

Estimating the actual costs of HAI as a measure of the cost savings attributable to prevention is difficult. Recent analyses estimated that the additional costs attributable to HAI were €5823–€11,840 ($7453–$15,155) per infected patient [4]. Previous reviews revealed that HAI causes annual financial losses of about €7 billion in Europe and about $6.5 billion in the USA, whereas the burden of HAI in developing countries was even higher [5].

Recent studies showed that patients aged 80 years, male, with longer hospital stay at the time of the survey, hospitalized in a critical care unit, with urinary catheter or central or peripheral catheter in place, who received mechanical ventilator support or underwent tracheotomy or hemodialysis during hospitalization, had an increased risk for HAI [6]. However, some HAI risk factors potentially suffer from a severe endogeneity bias. In particular, the direction of causality between incidence of HAI and length of stay is not well identified. One of the consequences of HAI consists in increasing the length of hospital stay, but a longer hospital stay increases the chances of getting an infection [7].

Despite their limitations, point prevalence surveys (PPS) are often preferred to prospective surveillance, since they provide a feasible estimate of HAI when resources are limited. Besides that, PPS usually represent the starting point to developing more comprehensive and detailed infection control programs [8]. However, only few surveys regarding HAI have been conducted in Italy and more information is needed.

The aim of this study was to analyze the results of a six-year point prevalence survey of HAI in a teaching acute care hospital in Italy and to investigate the main risk factors associated with them, with the purpose of focusing on specific HAI and planning targeted surveys in subsequent years to reduce the burden.

## 2. Materials and Methods

### 2.1. Study Design and Setting

Fondazione Policlinico Universitario A. Gemelli IRCCS (FPG) is a teaching acute care hospital that, in 2018, became Scientific Institute for Research and Healthcare. At that time, FPG had 1526 inpatient beds (1550 in 2016) and discharged 94,919 patients. In the same year (2018), 4110 children were born at FPG and 53,701 surgeries were performed [9].

A point prevalence survey to detect HAI was carried out in FPG for 6 years, from 2013 to 2018. The point prevalence survey took place in three successive days, between October and December, each year. The survey, promoted by the Hospital Infection Control Committee (CIO) involved all of Gemelli teaching Hospital structures and wards, both medical and surgical.

Inpatients of any age in FPG were eligible for inclusion. Patients in outpatient areas, emergency departments, rehabilitation units were excluded. Patients admitted for less than 48 h were also excluded, because this amount of time is considered as the “typical incubation period” [2] for HAI case definition. The wards’ nurses, from the morning census on the survey date, obtained all the eligible patients.

Information regarding conditions identified by scientific literature as risk factors for HAI were collected. In particular, the following were considered: surgical procedure occurring up to 30 days before survey; admission from nursing home, long-term care or dialysis centers in the previous 90 days; multidrug resistant (MDR) microorganism isolation in the previous 90 days; presence of urinary catheter or central or peripheral catheter, mechanical ventilator support or tracheotomy; type of antibiotic therapy; and the McCabe score, calculated to assess patient condition.

### 2.2. Training and Data Collection

Our survey was carried out by medical residents in Public health, Infectious Disease, Microbiology, and Occupational health. They were specifically trained for the task, as recommended by the European Centre for Disease Prevention and Control for point prevalence survey of healthcare-associated infections in European acute care hospitals [3].

A software, specifically developed for this objective, was used to collect data. Medical residents were adequately trained in data collection and system usage. The data collected for each patient included: age, sex, admission date of the patient in the hospital, current diagnosis of the disease, hospital ward for each patient, presence of invasive devices on the day of the investigation, and whether the patient had one or more HAI active and/or received antimicrobial treatment. Data regarding the site of infection, the date of HAI detection and pathogens that have been isolated were collected. Furthermore, information concerning the use of antimicrobial, type, purpose (therapeutic, prophylactic or therapeutic/prophylactic), and the results of blood cultures drawn before the use of such drugs were investigated as well. 

### 2.3. Case Definition

HAI was identified according to diagnosis guidelines from the ECDC in 2011 [3]. HAI primarily refers to infections acquired more than 48 h after admission. 

In this study, therefore, HAI was defined as an infection occurring from day 3 of hospital stay onwards and meeting the case definition on the day of the survey. The date of onset of HAI had to be considered as the date of first signs or symptoms of the infection or, if unknown, the date when antimicrobial treatment was started or the date when the first diagnostic sample was taken. However, surgical site infection met the criteria occurring from day 1 (day of admission) or day 2 if a surgical procedure was performed in the previous 30 days, or 90 if a prosthesis was placed. Likewise, an infection was classified as HAI if it occurred from day 1 or day 2 in a patient discharged from acute care hospital in the preceding 48 h or, for Clostridium difficile infection, in a patient discharged from acute care hospital in the preceding 28 days, or in a patient carrying an invasive device, inserted on this admission to hospital prior to onset.

HAI types of clinical and epidemiological relevance, that were specifically investigated in this point prevalence surveys, were surgical site infection (SSI), urinary tract infections (UTI), bloodstream infections (BSI), pneumonia, meningitis, and Clostridium difficile infections. The SSI was classified as defined by Centers for Disease Control and Prevention [10].

### 2.4. Statistical Analysis

A descriptive statistical analysis was performed using absolute and relative frequencies or mean and standard deviation (SD) for the following variables: age, gender, number of patients (total patients, number of patients grouped by location and number of patients with HAI), average length of stay at the moment of HAI detection, HAI prevalence, risk factors (mechanical ventilation, urinary catheter, central/peripheral catheter, surgical drain, antibiotics use, parenteral nutrition) and type of HAI (respiratory tract infections, surgical site infections, urinary tract infections, primary bloodstream infections, Clostridium difficile infections, and central nervous system infections).

T-test and Chi-square test were performed to highlight differences, respectively, between quantitative variables and qualitative variables.

As reported in Table 1, the differences between patients’ characteristics were tested between patients with HAI and overall patients. 

Multiple logistic regression was performed to evaluate the usefulness of specific independent variables in predicting the dichotomic dependent variable, HAI presence. The independent variables included in the model were sex, length of stay at the moment of HAI detection, age, surgical procedure up to 30 days before HAI diagnosis, immunodepression, use of mechanical ventilation, parenteral nutrition, presence of surgical drain, urinary catheter, central venous catheter (CVC), and antibiotic therapy.

A second multivariate logistic regression model was, subsequently, created, always considering HAI presence as dependent variable, with only length of stay at the moment of HAI detection, urinary catheter, CVC, and antibiotic therapy as independent variables. 

The statistical significance level was set at *p*-value <0.05 and all the analyses were carried out by using software Stata IC 13 for Mac” (Stata Corp, Lakeway, TX, USA).

## 3. Results

### 3.1. Patients

A total of 6263 patients were included in the 6 annual surveys. In detail, there were 904 patients enrolled in the survey of 2013, 907 in the survey of 2014, 1093 in 2015, 1131 in 2016, 1106 in 2017, and 1122 in 2018. The mean age was 57.86 ± 23.05 years (56.87 ± 23.00 for female and 58.88 ± 23.05 for male) and varied from 56.00 ± 24.31 in 2014 to 61.80 ± 17.52 in 2013; 50.84% of patients were female. Most patients were recruited from surgical (30.00%) and internal (41.02%) wards; 7.44% were recruited from critical care units. More than a quarter of all surveyed patients had a device in place on the survey date, had undergone an operative procedure during the current hospital admission, or had been previously admitted to the survey hospital within 3 months prior to the survey date (see in detail in Table 1). All the differences between patients grouped by location resulted to be statistically significant.

### 3.2. Prevalence and Distribution of Healthcare-Associated Infections

A total of 328 patients with HAI were detected during the entire survey, with a prevalence of 5.24% (95% CI: 4.70–5.83%), ranging from 3.16% in 2017 to 6.64% in 2013) (see in Table 2). 

Respiratory tract infections were the most common, followed soon after by surgical site infections, urinary tract infections, primary bloodstream infections, Clostridium difficile infections, and central nervous system infections (Table 3). All the differences between patients grouped by HAI type resulted to be statistically significant (*p*-value < 0.001) (Table 2).

The average length of stay at HAI detection time for all patients enrolled was 10.84 ± 18.06 day while the average length of stay at HAI detection time for patients without HAI was similar (10.05 ± 17.01 days), the average length of stay at HAI detection time for patients with HAI was far above (25.09 ± 27.97 days). The difference was statistically significant (*p*-value < 0.001).

### 3.3. Associated Risk Factors

The first logistic regression model, as described in detail in the Statistical analysis section, yielded an adjusted coefficient of determination (adjusted R^2^) of 0.2742. 

The second logistic regression model, as also described in detail in the Statistical analysis section, yielded a significant adjusted coefficient of determination (adjusted R^2^) of 0.2780. 

## 4. Discussion

In this survey 5.24% (95% CI: 4.70–5.83%) of patients, during the 6-year study period, had at least one HAI. The prevalence was slightly lower compared to the one previously reported from other European Countries (6.0%) [3] and the one reported from a recent USA study (6.0%) [11], but higher than the one from a Chinese report (3.7%) [6] and a cross-sectional Romanian study [12].

In accordance with other prevalence studies [6,13], males were more affected than females in this study, especially in relation to blood-stream and surgical site infections. This could be explained by a different propensity for skin colonization in relation to sex, while as pointed out by another study, the relationship between sex and the rate of HAI varied depending upon the underlying acute reasons for hospitalization [6].

The distribution of infection types in this study was similar to the one showed by the ECDC study, in which the highest prevalence of HAI was registered for pneumonia (1.3%) and SSI (1.3%) [3]. Unlike another study [12] that reported a high percentage of enterocolitis Clostridioides difficile, in our study, Clostridium difficile infections were not among the most frequent HAI. For these two conditions, however, the prevalence found in a study conducted in fifty-two Chinese hospitals was very different compared to our results [6]. This may be explained by different HAI case definitions adopted, as well as by different patient characteristics. In particular, the study conducted in fifty-two Chinese hospitals reported that only the 2.0% of enrolled patients were in critical care units, unlike the 7.4% of patients enrolled in our study.

The performed logistic regression analysis showed length of stay at the moment of HAI detection, urinary catheter, central venous catheter, and antibiotic therapy to be the most important predictors of HAI presence. The evidence to support an association between the presence of a urinary catheter or a CVC and the risk to acquire an HAI is widely reported [14,15,16,17]. For the antibiotic therapy, we surmise that antibiotics all result in a certain degree of normal flora depletion and this may explain in part why antibiotics are associated with greater risk in our study. The association between longer length of hospital stay and increased risk of HAI can largely be explained by the increased duration of stay among those who have underlying morbidity and require invasive procedures.

## 5. Study Limitations

This study has some limitations. 

First, if it is true that the determination of HAI, in the same period every year, allowed us to compare the results, avoiding the possible bias related to seasonal variation observed in several previous studies, the consequence is that we cannot know if the point prevalence is substantially different during other seasons of the year. Repeated point-prevalence surveys of HAI might be useful to trace the changes of HAI rates in different seasons.

Second, the study was monocentric. This limits the generalizability of the results. 

Third, the hospital size and the hospital type were not included as risk factors for HAI. Nevertheless, as reported in other studies [18], the patients admitted in large teaching hospitals are more likely to be patients with severe underlying medical conditions. In these hospitals, for instance, more complicated medical procedures are performed and that could increase the risk for HAI.

Despite the intrinsic limitations of prevalence investigations [19], for the first time, an international protocol was used in Liguria region (Italy) for a regional survey [20], allowing us to compare our results with those of similar studies conducted at a national and European level. Moreover, point prevalence studies, when periodically repeated, yield a great deal of information on the size of the phenomenon, highlighting the potential effects of the adopted strategies. Furthermore, data sharing should promote greater attention to the problem, at the local and regional level, and strengthen infrastructure and expertise to implement the surveillance. Additionally, the identification of common problems allows the establishment of shared priorities, and evaluation of the impact of the strategies.

The analyses of differences in the presence of risk factors were performed between all patients and patients with HAI. Even though two groups may be regarded as non-independent, the differences between the two can be approximated to the difference between patients with HAI and patients without HAI, given the low HAI prevalence.

The aforementioned study allows [20], in theory, to compare the results of this paper with those of a similar environment from an epidemiological standpoint (at a national and European level). Unfortunately, the results are not directly comparable due to the different study protocol used in the 2007 survey; therefore, this most recent experience should be considered as a starting point and further assessments will be possible when future surveillance data are available, according to the Regional Prevention Plan 2014–2018, which requires to carry out a prevalence survey every year.

## 6. Conclusions

Even though it is difficult to draw conclusions from the comparison with similar studies because of the differences in patient populations, case definitions, and data collection, HAI represents a substantial disease burden considering that, from 2013 to 2018, there were 6263 patients enrolled in this study, 328 of which acquired a healthcare infection (5.24%).

Similarly to other logistic regression analysis conducted on this topic, the length of stay at HAI detection time, antibiotic therapy, presence of urinary catheter or CVC resulted to be the most important predictors of HAI prevalence. This should encourage to introduce a proper prophylaxis program and to avoid exposure to these risk factors whenever possible.

In addition, ECDC provides a constant update of methods and results of periodically carried out point prevalence surveys [21] via an online database, point prevalence survey database HAI-Net, which represents an important frame of reference for future studies of this kind.

Finally, the results obtained from this point prevalence survey lead us to focus on surgical site infections. A 2-year incidence study to monitor them was, therefore, started in all of the surgical wards of our teaching hospital and is currently ongoing. In addition, a continuous incidence analysis of bloodstream infections and pneumonia occurring in the intensive care units of FPG from 2019 is ongoing. These incidence studies aim to control and reduce the impact of the most relevant and common infection among our patients.

## Figures and Tables

**Table 1:** Distribution of total patients and patients with healthcare-associated infections (HAI) stratified by gender, location, and associated risk factors.

Characteristics	Number of Patients (% of Total)	Patients with HAI (% of Total)	*p*-Value
Patient location			<0.001
Surgical wards Internal wards Paediatrics Geriatrics and rehabilitation Gynaecology and obstetrics Critical care unit	1879 (30.00%) 2569 (41.02%) 162 (2.59%) 344 (5.49%) 570 (9.10%) 466 (7.44%)	107 (5.69%) 129 (5.02%) 5 (3.09%) 16 (4.65%) 14 (2.46%) 50 (10.73%)	
Gender			<0.001
Male Female	3079 (49.16%) 3184 (50.84%)	191 (6.20%) 137 (4.30%)	
Risk factors			<0.001
Mechanical ventilator Urinary catheter Central/peripheral catheter Surgical drain Antibiotics use Parenteral nutrition	193 (3.08%) 1470 (23.47%) 979 (15.63%) 688 (10.99%) 2066 (32.99%) 264 (4.22%)	44 (22.80%) 192 (13.06%) 142 (14.50%) 66 (9.59%) 304 (14.71%) 52 (19.70%)	

**Table 2:** Distribution of the 328 healthcare-associated infections (HAI) stratified by year.

Year	HAI (% Prevalence)	95% Confidence Interval for % Prevalence
2013 2014 2015 2016 2017 2018	60 (6.64%) 48 (5.29%) 71 (6.50%) 62 (5.48%) 35 (3.16%) 52 (4.63%)	5.14–8.51% 3.97–7.01% 5.14–8.17% 4.26–7.01% 2.25–4.42% 3.51–6.08%

**Table 3:** Distribution of 328 healthcare-associated infections (HAI) stratified by type of infection.

Type of Infection	No. of Infections (% Prevalence)	% of Total HAI
Respiratory tract infections ^1^ Surgical site infections Urinary tract infections ^2^ Primary Bloodstream infections ^3^ Clostridium difficile infections Central nervous system infections	93 (1.48%) 89 (1.42%) 67 (1.07%) 65 (1.04%) 20 (0.32%) 14 (0.22%)	26.72% 25.57% 19.25% 18.68% 5.75% 4.02%

^1^ of which 25 (27% of all pneumonia) were associated with a mechanical ventilator; ^2^ of which 48 (72% of all urinary tract infections) were associated with a catheter; ^3^ of which 40 (62% of all bloodstream infections) were associated with a central venous catheter.
